# Solid Tumors and Kinase Inhibition: Management and Therapy Efficacy Evolution

**DOI:** 10.3390/ijms23073830

**Published:** 2022-03-30

**Authors:** Flávia Melo Cunha de Pinho Pessoa, Caio Bezerra Machado, Emerson Lucena da Silva, Laudreísa da Costa Pantoja, Rodrigo Monteiro Ribeiro, Maria Elisabete Amaral de Moraes, Manoel Odorico de Moraes Filho, Raquel Carvalho Montenegro, André Salim Khayat, Caroline Aquino Moreira-Nunes

**Affiliations:** 1 Department of Medicine, Pharmacogenetics Laboratory, Drug Research and Development Center (NPDM), Federal University of Ceará, Fortaleza 60430-275, Brazil; flaviamelop@outlook.com (F.M.C.d.P.P.); caio.bmachado97@gmail.com (C.B.M.); lucenaemerson@hotmail.com (E.L.d.S.); betemora@ufc.br (M.E.A.d.M.); odorico@ufc.br (M.O.d.M.F.); rcm.montenegro@gmail.com (R.C.M.); 2 Department of Pediatrics, Octávio Lobo Children’s Hospital, Belém 60430-275, Brazil; laudreisa@hotmail.com; 3 Department of Biological Sciences, Oncology Research Center, Federal University of Pará, Belém 66073-005, Brazil; khayatas@gmail.com; 4 Department of Hematology, Fortaleza General Hospital (HGF), Fortaleza 60150-160, Brazil; rmonteiroribeiro@icloud.com; 5 Northeast Biotechnology Network (RENORBIO), Itaperi Campus, Ceará State University, Fortaleza 60740-903, Brazil

**Keywords:** protein kinase inhibitors, TKIs, molecular targeted therapy, neoplasms

## Abstract

The increasing numbers of cancer cases worldwide and the exceedingly high mortality rates of some tumor subtypes raise the question about if the current protocols for cancer management are effective and what has been done to improve upon oncologic patients’ prognoses. The traditional chemo-immunotherapy options for cancer treatment focus on the use of cytotoxic agents that are able to overcome neoplastic clones’ survival mechanisms and induce apoptosis, as well as on the ability to capacitate the host’s immune system to hinder the continuous growth of malignant cells. The need to avert the highly toxic profiles of conventional chemo-immunotherapy and to overcome the emerging cases of tumor multidrug resistance has fueled a growing interest in the field of precision medicine and targeted molecular therapies in the last couple of decades, although relatively new alternatives in oncologic practices, the increased specificity, and the positive clinical outcomes achieved through targeted molecular therapies have already consolidated them as promising prospects for the future of cancer management. In recent years, the development and application of targeted drugs as tyrosine kinase inhibitors have enabled cancer treatment to enter the era of specificity. In addition, the combined use of targeted therapy, immunotherapy, and traditional chemotherapy has innovated the standard treatment for many malignancies, bringing new light to patients with recurrent tumors. This article comprises a series of clinical trials that, in the past 5 years, utilized kinase inhibitors (KIs) as a monotherapy or in combination with other cytotoxic agents to treat patients afflicted with solid tumors. The results, with varying degrees of efficacy, are reported.

## 1. Introduction

Cancer is reported by the World Health Organization as a leading cause of death worldwide among elderly populations. According to the World Health Organization, noncommunicable diseases (NCD), which include cancer as a major agent, are responsible for 71% of deaths worldwide every year and progress on the global goals for NCD prevention and control is still slow. As a clear barrier to life expectancy increases in the world, the cancer burden is expected to increase in the years to come and is estimated will afflict more than 28 million people in 2040 [1,2,3].

While incidence and mortality rates vary highly among different tumor subtypes, 18% of all cancer-related deaths in 2020 were attributed to lung tumors and lung, female breast, colon, stomach, liver, and esophagus cancers were, added together, responsible for approximately 50% of cancer mortality rates in the same year [3].

The increasing numbers of cancer cases worldwide and the exceedingly high mortality rates of some tumor subtypes raise the question about if the current protocols for cancer management are actually effective and what has been done in an effort to improve upon oncologic patients’ prognoses. In this study, we investigated clinical trials in the past 5 years that focused their efforts on kinase inhibitor (KI) treatment protocols after first-line treatment failure in solid-tissue cancers and we discussed the trends for popular molecular targets and KIs pharmacological characteristics.

## 2. Background of Cancer Management

As biological structures, tumors are highly dependent on the overexpression of cell proliferation and the survival mechanisms that sustain tumor growth, even in otherwise adverse scenarios. The malignant status of neoplastic clones is achieved through multifactorial events of normal human physiology, life habits, exposition to environmental agents, and genetic predispositions that together lead to failure in the DNA damage response (DDR) machinery and induce consequent DNA mutations and chromosomal abnormalities [4,5,6].

The traditional chemo-immunotherapy options for cancer treatment focus on the use of cytotoxic agents that are able to overcome neoplastic clones’ survival mechanisms and induce apoptosis, as well as on the ability to capacitate the host’s immune system to hinder malignant cells’ continuous growth [7,8,9]. Although considered milestones in the clinical management of oncologic patients, the above-mentioned therapies still struggle with the occurrence of severe adverse events because of their toxicity profiles over the homeostasis of healthy cellular populations [10,11,12].

Another major obstacle to the effectiveness of cancer management is the still highly dangerous emergence of multidrug resistance (MDR) cases, which are responsible for the majority of cancer relapses. MDR can be either intrinsic, existing inherently in a tumor even before treatment exposure, or acquired, emerging as a response of the neoplastic clones to the selective pressure of a drug’s cytotoxic activity, and both mechanisms can happen simultaneously and cooperate for malignant progression [13,14].

Regardless of being intrinsic or acquired, MDR pathways provide tumors with the ability to bypass the effects of proliferation and survival impairment imposed by cytotoxic treatments through mechanisms such as increased drug efflux caused by overexpression of the transmembrane transporters of the ATP binding cassette (ABC) family, upregulation of DDR proteins, epigenetic alterations modifying oncogene expression, and tumor microenvironment alterations [14,15].

The need to avert the highly toxic profiles of conventional chemo-immunotherapy and to overcome the emerging cases of tumor MDR has fueled a growing interest in the field of precision medicine and targeted molecular therapies in the last couple of decades. Although relatively new alternatives in oncologic practice, the increased specificity and the positive clinical outcomes achieved through targeted molecular therapies have already consolidated them as a promising prospect for the future of cancer management [15,16].

## 3. Kinase Activities and Inhibitors

Protein kinases (PK) are the main regulators of cell metabolism, being involved in pathways of cellular proliferation, survival, DNA repair, cytoskeleton organization, and cell cycle progression. This regulation takes place through PKs’ phosphorylation of serine, threonine or tyrosine residues in target proteins, altering their structural conformation and consequently inducing protein metabolic activation [17,18].

Structurally, PKs can be divided into either receptor kinases, proteins with a transmembrane domain that act as receptors for external growth and survival signals, and then become phosphorylate amino acid residues in the intracellular compartment, or non-receptor kinases, cytoplasmic or nuclear proteins that act as second messengers after prior activation by another intracellular signal [19,20].

Due to their major role in the regulation of cell signaling pathways, PK mutations and overexpression are well characterized as drivers of carcinogenesis. The most classic kinase associated with malignant phenotypes is the BCR activator of RhoGEF and GTPase—the ABL proto-oncogene 1 (*BCR-ABL*) chimeric protein that is formed through a reciprocal translocation between chromosomes 9 and 22 [18,21].

This cytogenetic abnormality, first observed in the early 1960s, is present in more than 90% of all chronic myeloid leukemia cases and fueled the development of imatinib mesylate, the first clinically available kinase inhibitor (KI) that, with its astounding rates of disease remission and mild side effects, roused an increased interest in targeting kinase inhibition in oncologic practices [22,23].

**Figure 1:**
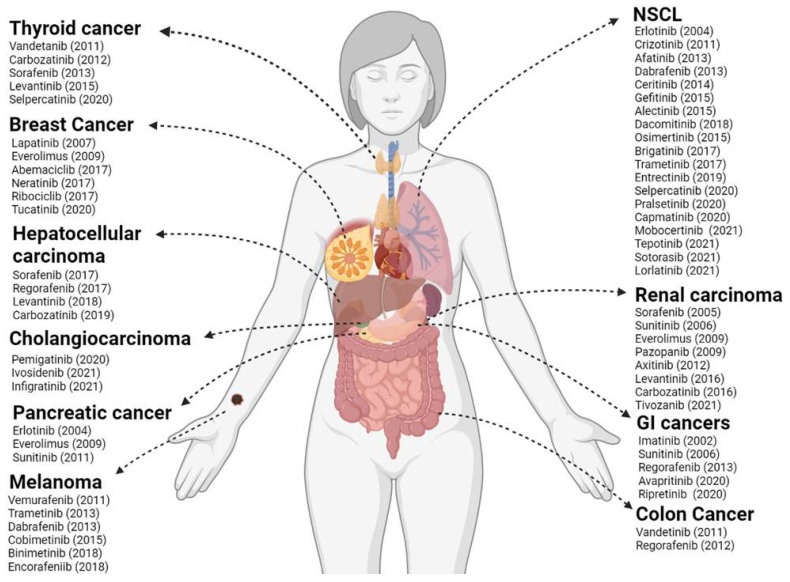
FDA-approved kinase inhibitors (KI) for solid tumor therapy. In the figure are listed the KIs and their respective year of approval by the FDA until 2022 in the therapy of the most relevant solid tumors in the clinic. NSCLC: non-small cell lung cancer; GI: gastrointestinal. Created with BioRender.com.

**Figure 2:**
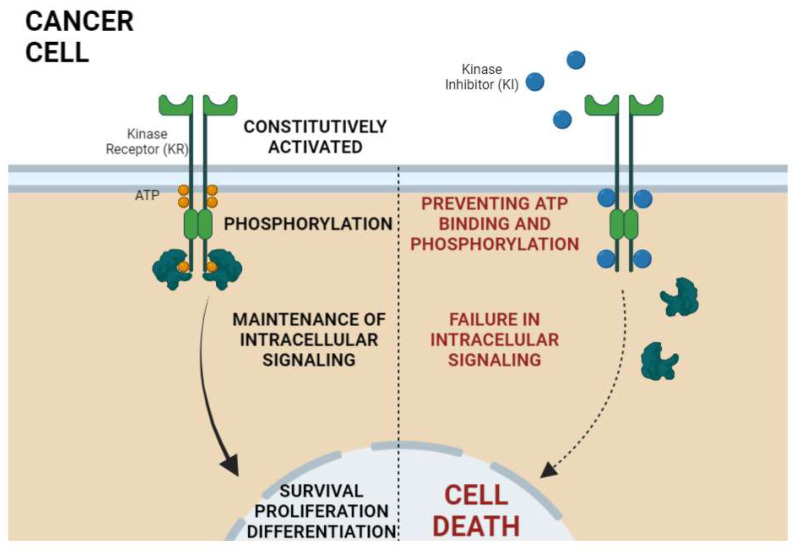
General mechanism of action of kinase inhibitors (KI) in cancer therapy. Kinase receptors (KR) are constitutively activated in cancer; that is, there is no need for extracellular ligands to lead to receptor activation. The KR activation is characterized by phosphorylation of intracellular protein domains of the receptor. Once phosphorylated, the propagation and maintenance of intracellular signaling by the activation of downstream proteins occurs, thus leading to the transcription of genes related to the malignant phenotype of cancer cells. In turn, the KIs bind at the ATP site via competitive inhibition, stopping cell proliferation signaling, which finally culminates in cell death. Created with BioRender.com.

Today, more than 70 KIs have received Food and Drug Administration (FDA) approval for cancer treatment (Figure 1) and about two dozen PKs are targets of inhibition among these treatment protocols. The mechanisms through which KIs inhibit kinase activity are diverse among different molecules and can be categorized into either reversible or non-reversible, also known as covalent, inhibitors (Figure 2). Reversible inhibitors are further stratified into categories I to V depending on the kinase conformation necessary for proper molecule interaction and their binding sites [24,25,26].

Even though targeted molecular therapies greatly enhance a cancer patient’s prognosis, impairments regarding kinase inhibition still need to be faced to achieve ideal outcomes in oncologic practices. Resistance cases dependent on kinase mutation or overexpression and acquired resistance pathways of increased drug efflux represent unavoidable obstacles that lead to the development of second and third generation KIs with increased kinase specificity and fewer off-target side effects [27,28].

The selection of the proper KI among the many different options available and understanding when to progress patients’ therapeutics from first- to second-line inhibitors are current challenges in the clinical practice and oncologic studies. Determining inhibitor selectivity and their outcomes in prognosis represent one of the major focuses for the advancement of present-day cancer-targeted molecular therapies [29,30].

## 4. Recent Prospects into Clinical Investigations

Usually, surgery is the most effective treatment for early-stage tumors, although most patients experience recurrence after radical surgery. In recent years, the development and application of targeted drugs have enabled cancer treatment to enter the era of specificity. In addition, the combined use of targeted therapy, immunotherapy, and traditional chemotherapy has innovated the standard treatment for many malignancies, bringing new light to patients with recurrent tumors [31].

**Figure 3:**
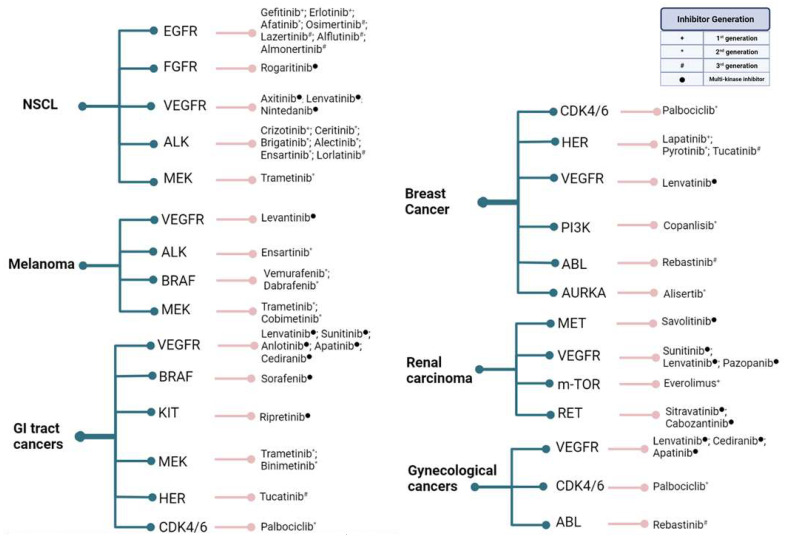
Common solid tumors that have been under active investigation in clinical trials for the efficiency of kinase inhibition over the past 5 years. Cancer types are followed by the targeted kinase and the inhibitors used. Gynecological cancers encompass ovarian, endometrial, and cervical tumors, while GI tract cancers encompass gastric, gastrointestinal, colorectal, pancreatic, biliary tract, and hepatocellular malignancies. As most novel inhibitors of growth factor receptors are developed to target multiple kinase pathways rather than specific emergent resistances, their denominations do not usually fit under the generational nomenclature and are addressed only as “multi-kinase inhibitors”. Created with BioRender.com.

Figure 3 exhibits a list of the most common solid tumors under active investigation in clinical trials for the efficiency of kinase inhibition over the past 5 years. While the number of studies investigating each tumor subtype varied highly, a consistency in aiming to evaluate next-generation inhibitors efficacy may be observed.

Imatinib, which was only released for use in 2001, is considered a milestone in the history of current medicine, as it is one of the main representants of the first generation of kinase inhibitors (KIs). Since it was developed, it has been possible to offer chronic myeloid leukemia patients a more effective therapy with fewer adverse events [32].

However, with prolonged use, patients show resistance to first generation KIs as tumor mutations that were able to evade their binding mechanisms began to emerge. Currently, several resistance mechanisms have been identified, such as amplification of the expression of target receptors, mutations in receptors that prevent KI binding, use of alternative pathways of cellular activation, and constitutive activation of downstream signaling effectors [33,34,35].

Therefore, second- and third-generation tyrosine kinase inhibitors were developed. These next-generation drugs are more selective to their targeted kinases and are able to intervene in a series of mutations that, until then, were not affected by KI therapies, making them much more potent and effective as a therapeutic option [35,36,37].

A clear example of the effectiveness of next-generation KIs was the accelerated FDA approval of osimertinib, a third-generation endothelial growth factor receptor (EGFR) inhibitor, for the treatment of EGFR-mutated non-small cell lung cancer (NSCLC). Prior to this approval, NSCLC therapeutics relied on the use of first-generation (erlotinib and gefitinib) and second-generation (afatinib and dacomitinib) EGFR inhibitors that would inevitably become inefficient because of the emergence of the EGFR T790M mutation [38,39].

Osimertinib molecular structure allows the inhibitor to covalently bind to T790M-mutated EGFR with much higher affinity than with wild-type EGFR, guaranteeing a treatment with milder side effects and more durable responses for NSCLC patients. Added benefits include its ability to trespass the blood-brain barrier and act upon brain metastases, which are a common topic of concern for patients afflicted with lung cancers [39,40,41].

While the astounding benefits over first-generation inhibitors granted osimertinib the status of a first-line treatment strategy in many EGFR-mutated NSCLC cases, this new alternative is still far from infallible. The molecular mechanism for inhibition of mutated EGFR by osimertinib requires its binding to a cysteine residue in the targeted kinase and the emergent mutation C797S, which changes the cysteine into a serine residue and is the new bottleneck for an improvement in patient prognosis [41,42].

Furthermore, a trend toward investigating anti-angiogenic inhibitors efficacy in all of the reported malignancy subtypes is also clear. Angiogenesis is considered a hallmark of cancer and is an essential process for tumor growth, nutrition, and oxygenation. Vascular endothelial growth factor receptor (VEGFR) inhibitors were the main focus of most anti-angiogenic approaches, with lenvatinib appearing as a proposed drug in all reported subsets. However, lenvatinib, as well as most other anti-angiogenic kinase inhibitors, has a multi-kinase activity, targeting other growth factor receptor pathways that may add to its efficacy in hindering malignant cell proliferation beyond only VEGFR inhibition, and its pharmacological characteristics will be discussed further ahead in this review [4,43,44].

Table 1 is comprised of a series of clinical trials with published results that, in the past 5 years, utilized KIs as a monotherapy or in combination with other cytotoxic agents to treat patients afflicted with solid tumors and results with varying degrees of efficacy were reported.

Of the 40 articles described in Table 1, 14 focused on patients afflicted with lung cancer and 6 focused on those afflicted with breast cancer. The other half of the articles analyzed studies focused on patients affected by various types of cancers, such as ovarian (3), renal (3), thyroid (2), colorectal (2), hepatocellular (2), cervical (1), urothelial (1), thymic (1), endometrial (1), nasopharyngeal (1), uterine (1) and bladder cancer (1). In total, 80% (32) of the articles described in the table are clinical trials of phase II and the other 20% (8) are composed of studies analyzing clinical trials of phase III.

A wide variety of KIs were described in the studies analyzed in Table 1. In order to facilitate the discussion of the table, only the 3 kinase inhibitors that were used most frequently will be discussed in depth, being Cabozantinib (17.5%), Lenvatinib (12.5%) and Buparlisib (7.5%).

### 4.1. Cabozantinib

Cabozantinib is a multi-kinase inhibitor of receptor tyrosine kinases hepatocyte growth factor receptor (MET), VEGFR family, and RET receptor tyrosine kinase (RET), among other carcinogenesis-related kinases. Since 2012, cabozantinib has accumulated U.S. Food and Drug Administration (FDA) indications for treatment of different malignancies and is currently recommended for management of advanced renal cell carcinoma (RCC), hepatocellular carcinoma (HCC), and adult and pediatric differentiated thyroid cancer (DTC) [26,85].

Molecularly, cabozantinib inhibits kinase activity through binding to ATP pockets in a reversible and competitive manner [86,87]. Its ability to inhibit multiple kinases, and consequently multiple cell signaling pathways, is an important aspect contributing to cabozantinib treatment success after previous failure with other VEGFR inhibitors because of emergent resistance mechanisms [88].

In hepatocellular carcinomas, inhibition of VEGFR alone is closely related with an increase in tumor metastasis potential caused by compensatory mechanisms of MET overexpression. Cabozantinib inhibition of both kinases is able to regulate tumor growth and invasiveness by hindering angiogenesis and promoting apoptosis, with evidence of reduction in metastasis focus after treatment [88,89,90].

Still to be fully elucidated is cabozantinib’s immunomodulatory activity over a tumor microenvironment and tumor-infiltrating macrophages and T cells. Contrasting data has been reported in the literature regarding MET inhibitors and programed cell death ligand 1 (PD-L1) expression, leaving it unclear if a synergetic effect of MET inhibition and disruption of PD-1/PD-L1 pathways may be relevant in the clinical practice [90,91].

In the aforementioned studies of Table 1, cabozantinib as a single agent was used as a strategy to treat patient cohorts of renal, urothelial, lung, ovarian, breast, and hepatocellular carcinomas. Achieved results were modest in most of the evaluated tumor subtypes, with overall response rates (ORR) varying from 10% to 20% of patients and partial responses representing the majority of cases [50,55,64,65,70,73,78].

The best rates of response were seen in patients afflicted with lung cancer that were previously screened for RET mutational status, a molecular target of cabozantinib, highlighting the prognostic significance of screening tumors for potential biomarkers of neoplastic importance before deciding on which kinase inhibitor is most appropriate for follow-up treatment [64].

Although ORRs are relatively low, cabozantinib activity still represents an improvement to the prognosis of treated patients because of the statistically significant clinical benefit ratio (complete responses + partial responses + stable disease) achieved in these studies, as well as the improvement on progression-free survival rates (PFS) and overall survival (OS) [50,55,64,65,70,73,78]. Results observed in the analyzed studies are comparable to those of previous clinical trials that ensured cabozantinib FDA approval for treating renal and hepatocellular carcinomas, thus pointing toward the inhibitor’s efficacy for further tumor subtypes [92,93].

Most adverse events (AE) reported across studies were low grade and manageable through dose reductions. The main AEs afflicting cabozantinib patients manifested as diarrhea, palmar-plantar erythrodysesthesia (PPE), fatigue, hypertension, and an increase in transaminase levels and, in general, seem to relate to cabozantinib activity over VEGFR and angiogenesis. Few major bleeding events were described, which have already been reported as relevant AEs in treatments with VEGFR inhibitors [50,55,64,65,70,73,78].

Overall, treatment with cabozantinib is demonstrated to be clinically beneficial to patients in a variety of tumor cohorts and to have a safely manageable toxicity profile when administered in the therapeutic doses.

### 4.2. Lenvatinib

Lenvatinib is a multiple receptor tyrosine kinase inhibitor that demonstrates potent antiangiogenic properties indicated as monotherapy or combination therapy for certain malignancies. Lenvatinib inhibits the kinase activities of VEGFR 1, 2, and 3, fibroblast growth factor receptors (FGFR) 1, 2, 3, and 4, platelet-derived growth factor receptor α (PDGFRα), RET, and KIT [94,95].

Tumor growth is dependent on the development and proliferation of new blood vessels. The inhibition of the VEGF receptors prevents tumor angiogenesis. Lenvatinib also has a direct inhibitory effect on tumor cell proliferation by blocking RET, PDGFR α, and KIT [4,94,96]. Lenvatinib’s mechanism occurs through its binding to the adenosine-triphosphate binding site of VEGFR2 and to a neighboring region via a cyclopropane ring and thereby inhibiting tyrosine kinase activity and associated signaling pathways [95].

A total of five studies utilized Lenvatinib as the main therapy for patients. In the studies discussed in Table 1, it was observed that Lenvatinib was used as a therapy for several types of cancer, including thymic carcinoma, lung adenocarcinoma, endometrial cancer, thyroid cancer, and hepatocellular carcinoma. Lenvatinib is FDA approved, for now, only for the treatment of radioactive iodine-refractory DTC, unresectable or advanced HCC, and advanced RCC [94].

The efficacy of lenvatinib varied little between the studies. Sato et al. had an ORR of 38% and a PFS of 9.3 months. Hida, et al. and Makker, et al. pointed to a PFS of 7.3 months and 7.4 months, respectively. Ikeda, et al. observed an OS of 18.7 months, while Wirth, et al. found a PFS of 18.8 months in patients using lenvatinib associated with a treatment for hypertension and 12.9 months in patients using only lenvatinib. When comparing with other studies that addressed the same types of cancer, it is possible to perceive similar data [61,74,76,79,80].

In a study made by Schulumberger, et al. about therapy with lenvatinib in patients with radioiodine refractory thyroid cancer, it was observed that the median PFS was 18.3 months with lenvatinib as compared with 3.6 months with [97].

Havel, et al. demonstrated in their study with 135 patients with non-squamous NSCLC, who had failed at least two prior treatments, that the median OS with lenvatinib plus best supportive care (BSC) was 38.4 weeks compared with 24.1 weeks in the placebo plus BSC group and that the median PFS was significantly prolonged in lenvatinib versus placebo recipients (20.9 vs. 7.9 weeks; *p*\0.001) [98]. Meanwhile, in the ongoing, open-label, phase II trial of Taylor, et al. in patients with metastatic or recurrent endometrial cancer it was observed that the median PFS was 5.4 months and the median OS was 10.6 months [99].

The most common treatment-related AEs observed in the studies were hypertension, PPE, nausea, diarrhea, decreased appetite, proteinuria, fatigue, headache, and hypothyroidism. This corroborates with data from other studies that indicate that the main AEs of any grade occurring in lenvatinib recipients are hypertension, diarrhea, fatigue or asthenia, decreased appetite, decreased bodyweight, nausea, vomiting, thyroid and cardiac dysfunction, PPE, and proteinuria [94,95,96].

### 4.3. Buparlisib

Buparlisib, formerly known as BKM 120, is an oral 2,6-dimorpholino pyrimidine derivative. It causes inhibition of phosphoinositide 3-kinase (PI3K) downstream signaling including downregulation of phosphorylated protein kinase B (p-AKT) and phospho-S6 ribosomal protein (p-S6R) [100,101].

The mechanism of action of buparlisib is binding to the adenosine triphosphate (ATP) binding cleft of the PI3K enzyme in a competitive manner. Buparlisib causes inhibition of wild-type and mutant PI3Kα isoforms and PI3K β, γ, and δ isoforms at nanomolar concentrations by an ATP-competitive approach. That way it can inhibit both the production of the secondary messenger phosphatidylinositol3,4,5-trisphosphate and the activation of the PI3K signaling pathway. This may result in inhibition of tumor cell growth and survival in susceptible tumor cell populations. Buparlisib is minimally effective against the PI3K class III family [100,102].

The studies in Table 1 that used buparlisib as a kinase inhibitor were all aimed at the treatment of breast cancer. Mutations in the PI3K pathway are frequent in breast cancer and also play a pivotal role in resistance to hormonal therapy and Her-2 targeted therapy [100]. This resistance can be associated with the activation of PI3K, AKT, and the mammalian target of the rapamycin (mTOR) pathway [103,104].

The medium PFS varied greatly between studies. The Di Leo, et al. and the Baselga, et al. studies utilized buparlisib associated with fulvestrant as treatment for patients of the positive group and compared results with those patients of the control group that received a placebo instead. Baselga, et al. demonstrated a median PFS of 6.9 months and 5 months in the buparlisib + fulvestrant-treated group and in the placebo-treated group, respectively. Meanwhile, Di Leo, et al. pointed to a median PFS of 8.3 months and 12 months in the buparlisib + fulvestrant group and in the placebo group, respectively. Garrido-Castro, et al. used only buparlisib to treat the patients who participated in the study, with a median PFS of 1.8 months and a median OS of 11.2 months [46,48,49].

When comparing with other studies, it is possible to notice that the median OS ends up being higher in the groups of patients treated with buparlisib, as demonstrated in the study by Campone, et al., in which the median OS was slightly higher in the buparlisib arm (33.2 months) versus the placebo arm (30.4 months) or even in the study of Soulieres et al., in which the median OS at data cut-off was 10 vs. 6.5 months for patients with head and neck squamous cell carcinoma treated with buparlisib + paclitaxel and placebo, respectively [105,106].

**Figure 4:**
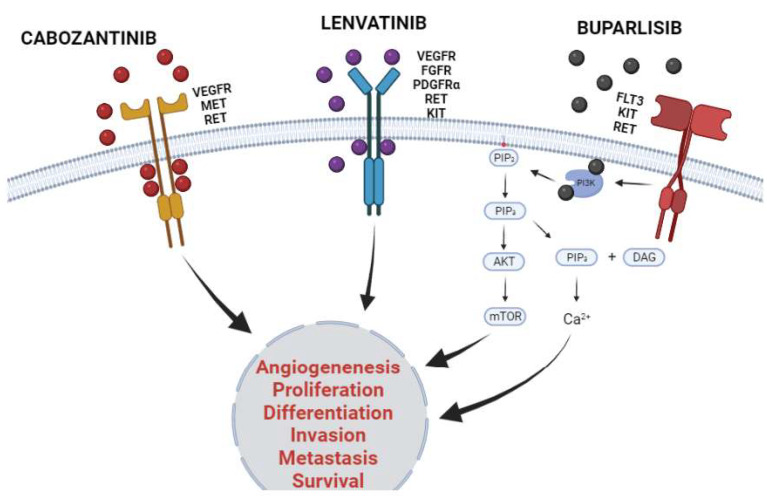
Mechanism of action of cabozantinib, lenvatinib, and buparlisib in cancer. The three KIs discussed were the most frequently studied in the last 5 years in monotherapy or in combination with other cytotoxic agents to treat patients afflicted with solid tumors. Cabozantinib and lenvatinib are multiple kinases inhibitors and have their inhibitory activity established in several families of KRs. Both KIs inhibit kinase activity through binding to ATP pockets reversibly and competitively, thus stopping downstream activation pathways. Otherwise, buparlisib inhibits the downstream enzyme phosphoinositide 3-kinase (PI3K) inhibiting PI3K/AKT/mTOR pathway and decreasing intracellular calcium concentration. The inhibitory activity of KI culminates in decreasing in malignant proliferative phenotype, as well as inhibits migratory profile and cancer survival. Created with BioRender.com.

All three studies reported hyperglycemia and an increase in hepatic transaminases (AST and ALT) as the main adverse effects related to the use of buparlisib [46,48,49]. These findings corroborate with data shown by other studies that determine that the most common adverse events noted with buparlisib are rash, hyperglycemia, derangement of liver functions, and psychiatric events, besides fatigue, nausea, and anorexia [107,108,109,110]. Figure 4 presented below shows representatively the mechanism of action of the three KIs discussed previously.

## 5. Conclusions

In summary, this review once again demonstrates the importance of using KIs for the treatment of solid tumors, considering that, in general, studies indicate better results in the treatment and quality of life of patients who use these therapies, either exclusively or associated with conventional therapies. It is important there be continuity in the studies on targeted therapies, aiming at higher rates of response and efficacy and, consequently, reducing toxicity and mortality rates observed in these patients.

## Figures and Tables

**Table 1:** Clinical trials utilizing kinase inhibitors (KI) as therapeutics for solid malignances in the past 5 years.

Study Phase	Cancer Type	Targeted Kinase	Kinase Inhibitor	Associated Treatment	Clinical Outcome	Adverse Events	References
II	Breast Cancer	PI3K	300 mg Alpelisib orally once per day	500 mg Fulvestrant intramuscularly on day 1 of each 28-day cycle and on day 15 of cycle 1	Median PFS was 7.3 months. Median overall survival was 17.3 months	The most frequent adverse events of grade 3 or more were hyperglycemia (28%), rash (9%), rash maculopapular (9%), and diarrhea (5%)	[45]
II	Breast Cancer	PI3K	Buparlisib at a starting dose of 100 mg once daily	NR	Median PFS was 1.8 months. Median OS was 11.2 months	The most frequently reported adverse events were fatigue (58%), nausea (34%), hyperglycemia (34%), and anorexia (30%)	[46]
II	Breast Cancer	HER-2	On days 1 through 21, patients of group 1 received oral Pyrotinib 400 mg once per day, and patients assigned to the group 2 received oral Lapatinib 1250 mg once per day	Patients in both groups also received oral capecitabine 1000 mg/m^2^ twice per day on days 1 through 14	The median PFS was 18.1 months in the Pyrotinib arm and 7.0 months in the Lapatinib arm	The most frequent grade 3 events were hand-foot syndrome (24.6% vs. 20.6%), diarrhea (15.4% vs. 4.8%), decreased neutrophil count (9.2% vs. 3.2%), and decreased WBC count (7.7% vs. 1.6%)	[47]
III	Breast Cancer	PI3K/AKT/mTOR	Buparlisib or Placebo (100 mg orally once daily starting on day 1 of cycle 1) in 28-day treatment cycles	Intramuscular injections of open-label Fulvestrant (500 mg on days 1 and 15 of cycle 1, and on day 1 of subsequent cycles)	Median PFS follow-up was 8.3 months in the buparlisib group and 12.0 months in the placebo group	The most frequent grade 3 adverse events were elevated ALT (18%), elevated AST (17%) and hyperglycemia (12%)	[48]
III	Breast Cancer	PI3K	Patients were randomly assigned (1:1) on day 15 of cycle 1 to receive either oral Buparlisib (100 mg once daily, starting from day 15 of cycle 1) or matching Placebo, starting on day 15 of cycle 1	Patients received intramuscular Fulvestrant 500 mg on days 1 and 15 of cycle 1, and on day 1 of subsequent 28-day cycles	Median PFS was 6.9 months in the Buparlisib group versus 5.0 months in the Placebo group	The most common grade 3–4 adverse events in the Buparlisib group versus the Placebo group were increased ALT (25% vs. 1%), increased AST (18% vs. 3%), hyperglycemia (15% vs. 1%)	[49]
II	Breast Carcinoma	MET	Cabozantinib at a daily oral dose of 100 mg during a 12-week, open-label, lead-in stage	NR	The estimated median overall PFS for all patients from study initiation was 4.3 months	The most common grade 3 events were PPE (13%) and fatigue (11%)	[50]
II	NSCLC	ALK	Lorlatinib 100 mg once daily (QD) was administered orally in 21-day cycles	NR	Median PFS was 6.6 months. Median OS was 20.7 months	The most frequently reported treatment-related AEs (all grades) were hypercholesterolemia (84.4%), hypertriglyceridemia (67.1%), edema (45.8%), peripheral neuropathy (34.2%)	[51]
II	NSCLC	ALK	Brigatinib at 90 mg once daily for the first 7 days and then at 180 mg once daily for cycle 1 (28 d per cycle)	NR	Median duration of response was 11.8 months. Median OS was not reached	The most common any-grade AEs were increased blood creatine phosphokinase (76%), diarrhea (43%), hypertension (40%), nausea (38%), increased lipase (33%), increased amylase (31%), increased AST (29%), and stomatitis (28%)	[52]
II	NSCLC	ALK	Brigatinib 90 mg once daily (arm A) or 180 mg once daily with a 7-day lead-in at 90 mg (arm B)	NR	Median PFS was 9.2 months in arm A and 15.6 months in arm B. Median OS was 29.5 months in arm A and 34.1 months in arm B	Most common any-grade AEs judged as related to treatment by the investigator were diarrhea (16% and 35%), nausea (26% and 33%), and increased blood creatine phosphokinase (14% and 32%)	[53]
II	NSCLC	ALK	Ensartinib 225 mg orally once daily on a continuous dosing schedule	NR	Median PFS in the full analysis set was 9.6 months. Median OS was not reached	The most common treatment-related adverse events were rash (56%), increased ALT (46%), increased AST (41%), increased creatinine (19%), constipation (18%) and pruritus (18%)	[54]
II	NSCLC	MET, VEGFR, AXL, ROS1, and RET	Cabozantinib at a daily oral dose of 100 mg during the 12-week open-label lead-in stage	NR	Median PFS post randomization was 2.4 months for Cabozantinib and 2.4 months for Placebo. The median OS for all patients from first dose of Cabozantinib was 7.7 months	The most common ≥ grade 3 events were fatigue (13%), PPE (10%), diarrhea (7%), hypertension (7%), and asthenia (5%)	[55]
II	NSCLC	EGFR	Osimertinib 80 mg orally once daily	NR	The median PFS was 9.9 months	The most common possibly causally related AEs were rash (42%), diarrhea (39%), dry skin (32%), and paronychia (32%)	[56]
II	NSCLC	ROS1	Crizotinib 250 mg orally twice daily on a continuous daily dosing schedule in 28-day cycles	NR	Median PFS was 15.9 months. Median OS was 32.5 months	The most frequently reported AEs of any grade were elevated transaminases (55.1%), vision disorder (48%), nausea (40.9%) and diarrhea (38.6%)	[57]
II	NSCLC	VEGFR	Fruquintinib 5 mg orally or matching Placebo. Treatment was given once daily in 4-week cycles	NR	The median follow-up for survival was 28.0 and 24.5 months in the Fruquintinib and Placebo groups, respectively	The most reported adverse events were hand-foot syndrome (49%), proteinuria (33%), blood TSH increased (28%), hoarseness (25%)	[58]
III	NSCLC	ALK	Ceritinib (750 mg orally per day, fasted, in continuous 21-day treatment cycles) or Chemotherapy (intravenous pemetrexed 500 mg/m^2^ or docetaxel 75 mg/m^2^ every 21 days)	NR	Median PFS assessed was 5.4 months versus 1.6 months	The most reported any-grade adverse events in the Ceritinib group were diarrhea (68%), nausea (58%), and vomiting (44%)	[59]
II	NSCLC	EGFR	Osimertinib 80 mg orally once daily	NR	Median PFS was 9.9 months	The most reported grade 1–2 adverse events were rash (40%), diarrhea (33%), dry skin (30%) and paronychia (26%)	[60]
II	Lung Adenocarcinoma	RET	Lenvatinib 24 mg orally once daily in 28-day cycles	NR	The median PFS was 7.3 months. The median OS was not reached	The most common any-grade AEs were hypertension (68%), nausea (60%), decreased appetite (52%), diarrhea (52%), proteinuria (48%), vomiting (44%), and headache (40%)	[61]
II	Alveolar soft-part Sarcoma	VEGFR, KIT and PDGFR	Cediranib or matching Placebo 30 mg orally once daily, for the first 24 weeks of the study	NR	12-month PFS was 38.7% for Cediranib and 34.4% for Placebo	The most common adverse events on blinded treatment were diarrhea (84%), hypertension (65%), fatigue (52%), and nausea (39%)	[62]
II	Squamous Cell Lung Cancer	PI3K	Taselisib was administered orally at 4 mg on an empty stomach in 21-day treatment cycles	NR	In the PAP, median PFS was 2.9 months and median OS was 5.9 months	The most reported adverse events were diarrhea (19%) and hyperglycemia (19%)	[63]
II	Lung Cancer	RET	Cabozantinib was administered in tablet form at a starting dose of 60 mg orally once daily	NR	The PFS was 5.5 months. The median overall survival was 9.9 months	The most common treatment-related adverse events of any grade were increased ALT (96%), increased AST (73%), hypothyroidism (69%), diarrhea (62%) and PPE (58%)	[64]
II	Renal Carcinoma	MET, VEGFR, RET, AXL, KIT and TIE-2	Cabozantinib 60 mg orally once daily in a fasted state	NR	The median PFS was not reached. The median OS was also not reached	The most frequently reported adverse events were PPE (62,9%), diarrhea (60%), hypertension (40%), proteinuria (40%), stomatitis (40%)	[65]
III	Renal Cell Carcinoma	VEGFR	Tivozanib 1.5 mg orally once daily in 4-week cycles comprising 21 days on treatment followed by 7 days off treatment or Sorafenib 400 mg orally twice daily continuously (with one cycle comprising 4 weeks of treatment)	NR	More responses were achieved in tivozanib group than sorafenib group and PR was the best response; Median PFS was 6.0 months in the Tivozanib group and 5.4 months in Sorafenib. Median OS was 16.4 months with Tivozanib and 19.7 months with Sorafenib.	The most common grade 3 or 4 treatment-related AE was hypertension (20% of patients treated with Tivozanib and 14% of patients treated with Sorafenib); Serious AEs occurred in approximately 10% of patients and were mainly gastrointestinal related; No deaths were attributed to treatment-related AEs	[66]
II	Renal Cell Carcinoma	VEGFR	Axitinib 5 mg twice daily taken orally	NR	Median PFS for all patients was 8.8 months	The most common adverse events of any grade were fatigue (83%), hypertension (75%), and hand-foot syndrome (65%)	[67]
II	Ovarian Cancer	VEGFR	Apatinib at an initial dose of 500 mg orally once daily	Oral Etoposide at a dose of 50 mg once daily on days 1–14 of a 21-day cycle	The median PFS was 8.1 months	The most common grade 3 or 4 adverse events were neutropenia (50%), fatigue (32%), anemia (29%), and mucositis (24%)	[68]
III	Ovarian Cancer	VEGFR	Pazopanib 800 mg or Placebo once daily for 24 months	NR	Median PFS was 18.0 months with Pazopanib and 23.9 months with Placebo	AE included hypertension (27%) and neutropenia (13%)	[69]
II	Ovarian Carcinoma	MET and VEGFR	Cabozantinib 100 mg orally and daily	NR	Median PFS after for Cabozantinib was 5.9 months compared with 1.4 months for Placebo	The most common grade 3 events were diarrhea (14%), PPE (6%), asthenia (6%), hypertension (6%) and neutropenia (6%)	[70]
II	Colorectal Cancer	VEGFR	Apatinib in a daily dose of 500 mg	NR	The median PFS of the whole group was 3.9 months. The median OS was 7.9 months	The common side effects of Apatinib were hypertension (76.9%), hand-foot syndrome (11.5%), proteinuria (73%), and diarrhea (23%)	[71]
III	Colorectal Cancer	VEGFR	Nintedanib 200 mg orally twice daily or matching Placebo twice daily in 21-day courses	BSC	Median OS was 6.4 months with Nintedanib and 6.0 months with placebo. Median PFS was 1.5 versus 1.4 months	The most frequent grade 3 AEs in the Nintedanib group were liver-related AEs, mainly increased ALT (8%) and AST levels (8%)	[72]
II	Hepatocellular carcinoma	MET	Cabozantinib at a starting dose of 100 mg daily during a 12-week. At week 12, patients with SD were randomized to Cabozantinib or Placebo, patients with a PR could continue open-label Cabozantinib treatment, and patients with PD at or before week 12 discontinued treatment	NR	Median PFS from time was 2.5 months for Cabozantinib patients and 1.4 months for Placebo	The most common grade 3 AEs were diarrhea (20%), hand-foot syndrome (15%), thrombocytopenia (15%), hypertension (10%), and transaminase elevation (10%)	[73]
II	Hepatocellular Carcinoma	VEGFR	Levantinib 12 mg orally and daily in 28-day cycles	NR	Median OS was 18.7 months.	The most common any-grade AEs were hypertension (76%), PPE (65%), decreased appetite (61%), and proteinuria (61%)	[74]
II	Thyroid Cancer	VEGFR	VEGF Trap given at a starting dose of 4 mg/kg every 2 weeks	NR	The median OS was 13.9 months	The most common grade 3/4 toxicities related to VEGF Trap were proteinuria (17%) and hypertension (12%)	[75]
III	Thyroid Cancer	VEGFR, RET, KIT, PDGFR and FGFR	Oral Lenvatinib (24 mg once daily) or placebo in 28-day cycles	NR	The median PFS for Lenvatinib-treated patients with and without TE-HTN was 18.8 months and 12.9 months, respectively	Arrhythmias (22%), congestive heart failure (28%), coronary artery disease (6%), peripheral vascular events (2%) and stroke (6%)	[76]
II	Cervical Cancer	HER-2	Neratinib 240 mg once daily with food on a continuous basis	Loperamide prophylaxis during cycle 1 (12 mg/day on days 1–14; 8 mg/day on days 15–28)	ORR was 25% and patients with PR had reduction in tumor size > 50%; Median PFS was 7.0 months and median OS was 16.8 months	Diarrhea (75%), nausea (44%), and decreased appetite (38%) were the most common adverse events	[77]
II	Urothelial Carcinoma	VEGFR, MET, AXL and RET	Cabozantinib was administered orally at a dose of 60 mg per day for 28 consecutive days	NR	In cohort one, the only non-exploratory cohort, ORR was 19.1%, median PFS was 3.7 months and median OS was 8.1 months; 45.2% of patients had SD as best response	Treatment related AEs were mild to moderate and included PPE (83%), anemia (79%), fatigue (69%), diarrhea (67%) and AST increase (59%) The most common treatment-related grade 3–4 AE was fatigue (9%)	[78]
II	Thymic Carcinoma	VEGFR, FGFR, RET and KIT	24 mg of Lenvatinib orally once daily in 4-week cycles	NR	ORR was 38% with all cases of response being PR; 57% of patients had SD; Median PFS was 9.3 months	The major treatment-related AEs were hypertension (88%), decreased platelet count (52%), diarrhea (50%), and PPE (69%)	[79]
II	Endometrial Cancer	VEGFR	20 mg oral Lenvatinib daily	200 mg intravenous pembrolizumab every 3 weeks	The median PFS was 7.4 months	Overall, the most frequently reported any-grade treatment-related adverse events were hypertension (25%), fatigue (49%), diarrhea (43%), and hypothyroidism (47%)	[80]
II	Nasopharyngeal Carcinoma	VEGFR	Axitinib at starting dose of 5 mg twice-daily taken orally with food in 4-week cycles continuously	NR	The median time-to-progression and PFS were both 5 months. The median OS was 10.4 months	The most common treatment-related AEs were hand-foot syndrome (50%), hypothyroidism (50%), fatigue (40%), hypertension (38%) and diarrhea (33%)	[81]
II	Gastrointestinal stromal turmour	KIT	Dovitinib was administered orally (500 mg/day, 5 days on/2 days off), and was taken either with or without food	NR	The median PFS was 4.6 months. The median OS was not reached	The most frequently observed grade 3 adverse effects were hypertension (17.9%), fatigue (12.8%), vomiting (10.3%), Y-Glutamyl transferase increase (10.3%)	[82]
II	Uterine Leiomyosarcoma	AURKA	Alisertib 50 mg twice daily by mouth on days 1–7 of each 21-day cycle	NR	The median PFS was 1.7 months and median overall survival 14.5 months	The most reported grade 1–2 adverse events included: thrombocytopenia (42.8%) and anemia (57.1%)	[83]
III	Bladder Cancer	EGFR and HER-2	Lapatinib was administered continuously at 1500 mg once daily (six 250-mg tablets). In the Placebo group six visually identical tablets were administered instead	NR	The PFS for Lapatinib and Placebo was 4.5 months and 5.1 months, respectively	The most reported adverse events diarrhea (60.8%), fatigue (35.1%), nausea (22.7%), rash (44.3%), pain (38.1%) and infection (26.8%)	[84]

NSCLC: non-small cell lung cancer; PI3K: phosphatidylinositol-4,5-bisphosphate 3-kinase; ALK: anaplastic lymphoma kinase; NR: not reported; PFS: progression-free survival; OS: overall survival; AE: adverse events; PPE: Palmar-plantar erythrodysesthesia; PDGFR: platelet-derived growth factor receptor; TSH: thyroid-stimulating hormone; AST: aspartate aminotransferase; ALT: alanine aminotransferase; MET: mesenchymal epithelial transition; VEGFR: vascular endothelial growth factor receptor; TIE-2: angiopoietin receptor; HER-2: human epidermal growth Ffctor receptor-type 2; ORR: objective response rate; FGFR: fibroblast growth factor receptors; PR: partial response; EGFR: epidermal growth factor receptor; AURKA: aurora kinase A; m-TOR: mammalian target of rapamycin; WBC: white blood cells; TE-HTN: treatment-emergent hypertension; SD: stable disease; RET: rearranged during transfection; KIT: KIT proto-oncogene; AXL: AXL receptor tyrosine kinase; ROS1: ROS proto-oncogene 1; BSC: best supportive care.
